# Correlation of attention deficit hyperactivity disorder with gut microbiota according to the dietary intake of Korean elementary school students

**DOI:** 10.1371/journal.pone.0275520

**Published:** 2022-09-30

**Authors:** Tae-Hwan Jung, Hyo-Jeong Hwang, Kyoung-Sik Han

**Affiliations:** Department of Food and Nutrition, Sahmyook University, Seoul, Korea; University of Nebraska-Lincoln, UNITED STATES

## Abstract

We investigated the impact of dietary patterns on the gut microbiota and concentration of short-chain fatty acids in the feces of Korean elementary school students. The dietary intake and ADHD assessment of 40 Korean elementary school students were analyzed using a dish-based semi-quantitative food frequency questionnaire. Analysis of gut microbiota and short-chain fatty acids composition were performed using the real-time polymerase chain reaction, metagenomics, and gas chromatography methods. The dietary patterns of participants were divided into four groups: healthy, processed food, fish and shellfish, and meat. The participants were also divided into two groups according to their ADHD scores: 0–30, control group; over 30, ADHD group. The ADHD score of the processed food group was significantly higher than that of the healthy group. The processed food and ADHD groups showed significantly higher abundance of harmful bacteria, such as the *Enterobacter*, *Escherichia coli*, and *Clostridium* strains, and markedly lower abundance of beneficial bacteria, such as the *Bifidobacterium* and *Ruminococcus* strains, than the control group. The heat maps of metagenomics indicated that each group was separated into distinct clusters, and the processed food and ADHD groups showed significantly lower α-diversity of gut microbiota than the control group. In these groups, the concentration of acetate or butyrate in the feces was significantly lower than that in the control group. These results may indicate that imbalanced diets can disturb the colonic microbial balance and are likely to become a potential risk factor for the prevalence of ADHD.

## Introduction

Attention deficit hyperactivity disorder (ADHD) is one of the most common mental disorders in childhood [1]. It is a neurodevelopmental disorder that leads to a failure in the control of one’s own behavior, causing various complications, such as depression, anxiety, and the bipolar disorder [2–4]. ADHD symptoms appear during childhood, being recognized as a medical condition, and tend to persist in adulthood [5]. ADHD is not only associated with neurotransmitters, like dopamine, but also with the immune system, which is greatly affected by the alteration of the gut microbiota composition [6, 7].

The genetic information of gut microbiota is known as the second genome of humans and several studies have reported that its abnormal alteration is associated with various diseases [8–10]. The interaction between the brain and gut is bidirectional communication because the gut is connected to the brain via 200–600 million neurons [11]. The gut microbiota can affect the enteric nervous system (ENS), which interacts with the central nervous system (CNS) of the brain [12]. The balanced gut microbiota composition contributes to health promotion, while its abnormal state can result in mental disorders by adversely affecting the ENS and CNS [13]. Therefore, the desirable modulation of gut microbiota may prevent and improve mental disorders [14]. Recently, studies have shown that the gut microbiota is associated with ADHD symptoms [15], which are related to the levels of neurotransmitters, such as dopamine and serotonin [16, 17]. Moreover, it was reported that beneficial bacteria, such as the *Bifidobacterium* strains, are associated with the increase of a dopamine precursor, indicating that the gut microbiota and their metabolites may modulate the release of neurotransmitters related to ADHD [18].

The composition of the gut microbiota is affected by various factors, such as region, race, and diet [19]. Dietary intake may have an especially important effect on the gut microbiota [20], the composition of which may be related to the short-chain fatty acids (SCFAs) produced by the metabolism of dietary ingredients [21]. SCFAs can modulate the intestinal environment to facilitate the growth of beneficial bacteria, such as the *Lactobacillus* and *Bifidobacterium* strains, resulting in the prevention and improvement of diseases through a balanced composition of the gut microbiota [22]. SCFAs can affect the brain function and the gut-brain interactions via direct or indirect pathways [17]. Alterations in the gut microbiota composition according to dietary intake are interrelated with health and disease [23, 24]. Millichap and Yee [25] revealed that the western dietary pattern is related to ADHD symptoms owing to the higher intake of saturated and refined sugars. Moreover, high intake of processed foods and snacks is associated with the aggravation of ADHD [26]. Therefore, this study aimed to investigate the correlation between the alteration of the gut microbiota composition and ADHD according to the dietary intake of Korean elementary school students.

## Methods

### Dietary intake investigation

This study was approved by the Sahmyook University Ethics Committee (2-7001793-AB-N-012018037HR). The dietary intake of 40 Korean elementary school students (11 years old) was analyzed using the Can Pro 4.0 (The Korean Nutrition Society, Seoul, Korea) database and a dish-based semi-quantitative food frequency questionnaire (FFQ), which was filled out by the parents. Based on the frequency of intake, the participants were divided into nine categories (more than three times a day, twice a day, once a day, five to six times a week, three to four times a week, one to two times a week, two to three times a month, once a month, and almost none), and based on the amount of intake, they were divided into three categories (usually less than normal, normal, and more than normal). The FFQ consists of 107 food items commonly consumed by Korean school-aged children. These foods are classified into 30 major groups by slight modifications to the method described by An et al. [27]: rice, 2 items; noodles, 2 items; instant noodles, 1 item; dumplings, 1 item; cereal, 1 item; rice cake, 1 item; bread, 2 items; fats, 2 items; fast foods, 3 items, Korean snacks, 3 items; potato, 1 item; sweet potato, 1 item; beef, 6 items; pork, 3 items; processed meat, 1 item; chicken, 3 items; egg, 1 item; fish, 8 items; shellfish, 4 items; fish cake, 1 item; milk/dairy foods, 6 items; beans, 5 items; vegetable, 18 items; mushroom, 1 item; seaweeds, 2 items; sweets, 7 items; nuts, 1 item; fruits, 9 items; beverage, 7 items; and kimchi, 4 items (Table 1). Dietary patterns were identified using principal component factor analysis, referring to the method of An et al [27] and Woo et al. [28]. The factors to be maintained for the division of dietary patterns were determined by the criteria of eigenvalues (above 1.0) and were rotated using a varimax procedure to achieve a structure with greater interpretability. Food groups with factor loading scores over 0.5, which represent the correlation coefficients between the individual food groups and dietary patterns, were considered to be the primary contributors.

**Table 1:** Lists of food items in a dish-based semi-quantitative food frequency questionnaire.

Food groups	Number	Food items
Rice	2	White rice, multi-grain rice
Noodles	2	Noodle soup, noodles
Instant noodles	1	Instant noodles
Dumpling	1	Dumpling
Cereal	1	Cereal
Rice cake	1	Rice cake
Bread	2	Bread, red bean bread
Fats	2	Butter, mayonnaise
Fast foods	3	Pizza, hamburger, fried potatoes
Korean snacks	3	Gimbab, stir-fried rice cake, Korean sausage
Potato	1	Potato
Sweet potato	1	Sweet potato
Beef	6	Bulgogi, roast beef, roasted ribs, short rib soup, soy sauce braised beef, beef tripe
Pork	3	Grilled pork belly, pork cutlet, pork slices,
Processed meat	1	Ham
Chicken	3	Fried chicken, chicken stew, ginseng chicken soup
Egg	1	Egg
Fish	8	Fried white flesh fish, baked white flesh fish, fried external blue-coloured fish, etc.
Shellfish	4	Clam, squid, shrimp, crab
Fish cake	1	Fish cake
Milk/dairy foods	6	Milk, low fat milk, process milk, yogurt, fermented milk, cheese
Beans	5	Bean boiled in soy sauce, bean curd, soybean paste soup, soy milk, soybean paste
Vegetables	18	Lettuce, perilla leaf, cucumber, carrot, chili, garlic, onion, spinach, water parsley, a green pumpkin, etc.
Mushroom	1	Mushroom
Seaweeds	2	Laver, seaweed
Sweets	7	Ice cream, candy, chocolate, snack, soda, cocoa, coffee
Nuts	1	Nuts
Fruits	9	Strawberry, apple, orange, pear, banana, watermelon, melon, grape, peach
Beverages	7	Orange juice, tomato juice, fruit juice, tea, diet drinks, coffee, sports drink
Kimchi	4	Kimchi, kimchi stew, diced radish kimchi, water kimchi

### ADHD assessment

We estimated the ADHD scores of participants using Korean ADHD rating scale (K-ARS), which is commonly used in various institutions to assess the ADHD scores of children in Koreans. K-ARS, which was filled out by parents and class teachers, uses a four-point scale (0 = never, 1 = sometimes, 2 = often, 3 = very often) and consists of 18 questions, of which nine each are related to the attention deficit and hyperactivity disorders, respectively. The ADHD group was identified using the criteria of Lee [29] and the participants were divided into two groups according to their ADHD scores: 0–30, control group; above 30, ADHD group.

### Anthropometric characteristics and sampling

We investigated the height, weight, body mass index (BMI), waist-to-hip ratio, and blood pressure of the participants. We also recorded the disease and medication history of the candidates and collected their feces to analyze the gut microbiota composition and fecal SCFA concentration.

### Quantitative real-time polymerase chain reaction (qPCR)

qPCR was performed using DT-prime 5 (DNA-Technology, Moscow, Russia) with the power SYBR Green PCR master mix (Applied Biosystems, Foster City, CA, USA) and specific primers to measure the total number of bacteria (forward, 5′-GCAGGCCTAACACATGCAAGTC-3′; reverse, 3′-CTGCTGCCTCCCGTAGGAGT), *Bifidobacterium* (forward, 5′-CTCCTGGAAACGGGTGG-3′; reverse, 3′-GGTGTTCT-TCCCGATATCTACA-5′), *Lactobacillus* (forward, 5′-CGATGAGTGCTAGGTGTTG-GA-3′; reverse, 3′-CAAGATGTCAAGACCTGGTAAG-5′), *Clostridium* (forward, 5′-ATGCAAGTCGAGCGAKG-3′; reverse, 3′-TATGCGGTATTAATCTYCCTTT-5′), *Enterobacter* (forward, 5′-ATGGCTGTCGTCAGCTCGT-3′; reverse, 3′-CTACTTCT-TTTGCAACCCACTC-5′), and *E*. *coli* (forward, 5′-GTTAATACCTTTGCTCATTGA-3′; reverse, 3′-ACCAGGGTATCTAATCCTGTT-5′) strains based on the method described by Han et al [30]. The reaction mixture consisted of 10 μL SYBR green master mix, 1 μL each of specific primer (10 pM), and 2 μL DNA sample to make a final volume of 20 μL. An initial DNA denaturation step at 95°C for 10 min was followed by 40 cycles of amplification (95°C for 15 s, primer annealing at 55–61°C for 25 s, and extension at 72°C for 30–40 s) and cooling at 4°C. The calibration curve was constructed using Ct values depending on the serial dilutions of bacterial DNA isolated from each bacterial strain using the DNeasy Blood & Tissue Kit (Qiagen, Hilden, Germany).

### Metagenomics analysis

The composition of the gut microbiota was determined at ChunLab Inc. (Seoul, Korea). PCR amplification was performed using fusion primers targeting the V3 to V4 regions of the *16S rRNA* gene in the extracted DNA. For bacterial amplification, fusion primers 341F (5′-TGATACGGCGACCACCGAGATCTACACXXXXXXXXTCGTCGGCAG-CGTCAGATGTGTATAAGAGACAGCCTACGGGNGGCWGCAG-3′; underlined sequence indicates the target region primer) and 805R (5′-CAAGCAGAAGACGGCA-TACGAGAT-X-GTCTCGTGGGCTCGGAGATGTGTATAAGAGACAGACTACH-VGGGTATCTAATCC-3′). PCR was conducted using Master Mix and PTC-200 Peltier Thermal Cycler (Applied Biosystems, Foster City, CA, USA) using the following program: initial denaturation step at 94°C for 5 min; 30 cycles of denaturation at 94°C for 30 s, annealing at 55°C for 45 s, and extension at 72°C for 90 s. Each PCR product was purified using a QIAquick PCR Purification Kit (Qiagen, Hilden, Germany) and the refined PCR products were quantified using the Quant-iTTM PicoGreen dsDNA Assay Kit (Invitrogen, Carlsbad, CA, Austria). Each DNA sample was mixed in the same amount and electrophoresis was performed on the pooled DNA sample. After emulsion-based clonal amplification of the DNA library samples, sequencing was performed using the emPCR Amplification 7020 Thermal cycler (Applied Biosystems) using the following program: initial denaturation step at 94°C for 4 min; 50 cycles of denaturation at 94°C for 270 s, annealing at 58°C for 45 s, and extension at 68°C for 30 s. Data analysis was performed using the CLcommunity^TM^ v.3.46 software (ChunLab Inc., Seoul, Korea) and the EZBioCloud database.

### Measurement of fecal SCFAs

Fecal SCFAs were measured using gas chromatography. Dried feces (10 mg) were mixed with 1 mL of methanol (Sigma, Saint Louis, MO, USA) and then shaken at 200 rpm for 90 min at 25°C. After the mixture was centrifuged at 10,000 rpm for 10 min at 25°C, the supernatants were filtered through a 0.22 μm syringe filter (Advantec, Tokyo, Japan), and 2 μL of the filtrate was injected into YL 6100GC (Youngin Chromass, Gyeonggi-do, Korea) to analyze the SCFA content in the colonic digest. Calibration curves were obtained using standard reagents (Sigma-Aldrich, St. Louis, MO, USA).

### Statistical analysis

The results are presented as the mean ± standard error of the mean (SEM). The significance of the differences among the groups was determined using the SAS/PROC GLM software (SAS v.9.1; SAS Institute Inc., Cary, NC, USA). The statistical significance according to dietary pattern was analyzed by one-way analysis of variance (ANOVA) with Duncan’s multiple range test. Statistical significance depending on the ADHD score was analyzed using Student’s t-test. Correlation analysis was used to analyze the relationship between the ADHD scores, intestinal microorganisms, and SCFAs according to the dietary patterns using the Pearson correlation coefficient.

## Results

### Dietary patterns

The dietary intake of the participants was investigated using FFQ. Factor analysis was performed to distinguish dietary patterns, and primary factors were separated through principal axis extraction and the varimax rotation method. As shown in Table 2, the four dietary patterns (healthy, n = 12; processed food, n = 8; fish and shellfish, n = 11; and meat, n = 9) were distinguished based on the foods or food groups with factor loading scores with absolute values over 0.5, according to the method of An et al. [27] and Woo et al. [28] and which reported the association between ADHD and dietary patterns. The "healthy" dietary pattern included high intakes of kimchi, fruits, vegetable, sweet potato, milk and dairy foods, and mushroom. The "processed food" dietary pattern was characterized by high intakes of fats, sweets, and fast foods. The "fish and shellfish" dietary pattern was identified by high intakes of shellfish, fish, and fishcake. The "meat" dietary pattern included high intakes of beef and pork. The four dietary patterns accounted for 44.65% of the variance in food intake, and each dietary pattern explained 14.39%, 10.09%, 12.09%, and 8.08% of the variation in food intake, respectively.

**Table 2:** Factor loadings for the major dietary patterns derived from the principal components analysis with orthogonal rotation.

Foods/Food groups	Dietary patterns			
	Healthy (n = 12)	Processed food (n = 8)	Fish and shellfish (n = 11)	Meat (n = 9)
Rice	.250	-.214	.099	-.066
Noodles	.221	.406	.215	.044
Instant noodles	.406	.338	.135	-.309
Dumpling	.338	.422	.436	.263
Cereal	-.102	-.095	.337	.493
Rice cake	.319	.051	.078	.465
Bread	.305	-.016	-.032	.026
Fats	-.104	**.680**	.076	.049
Fast foods	.039	**.638**	-.083	.149
Korean snacks	.400	-.003	.314	.053
Potato	.019	.320	.487	-.051
Sweet potato	**.583**	.294	.161	.165
Beef	.027	-.084	.010	**.677**
Pork	.095	.369	.230	**.596**
Processed meat	.397	.385	.479	.094
Chicken	.409	.102	.452	.134
Egg	.413	-.069	.139	.160
Fish	.377	.004	**.794**	.208
Shellfish	.142	-.078	**.923**	-.069
Fishcake	-.080	.444	**.590**	.165
Milk/dairy foods	**.544**	.044	.098	.325
Beans	.324	.293	-.079	.437
Vegetables	**.622**	.441	.190	.172
Mushroom	**.512**	.235	-.243	-.004
Seaweed	.481	.192	-.222	.489
Sweets	.353	**.679**	.192	-.080
Nuts	.438	.039	.203	.004
Fruits	**.647**	.180	.165	-.015
Beverages	.068	.274	.027	.392
Kimchi	**.717**	-.036	.514	.266
Variance explained (%)	14.39	10.09	12.09	8.08

Dietary patterns were determined using a factor analysis. The values in bold refer to the factor loadings of over 0.5

### ADHD Score and characteristics of participants

The ADHD score of participants was investigated using the K-ARS, which was examined by the parents and class teachers, using a four-point scale and consisted of 18 questions, containing nine questions each related to the attention deficit and hyperactivity disorders, respectively. ADHD scores according to the four dietary patterns are as follows: healthy group, 10.04 ± 2.83; processed food group, 21.44 ± 4.74; fish and shellfish group, 13.73 ± 3.82; meat group, 15.56 ± 4.23. No significant difference in ADHD scores was noted among the healthy dietary pattern, meat dietary pattern, and fish dietary pattern, whereas those of processed food dietary pattern were remarkably (*p* < 0.05) higher than those of the healthy dietary pattern. The ADHD group was identified using the criteria of Lee [29] and the participants were divided into two groups depending on their ADHD scores: 0–30, control group (n = 33); above 30, ADHD group (n = 7). In this study, we investigated the characteristics of participants, such as sex, age, BMI, parental smoking, parental income (Table 3). However, the basic characteristics did not significantly relate to the dietary patterns and ADHD scores.

**Table 3:** The characteristics of participants.

	Number	Percentage (%)
Sex (n = 40)		
Male	26	65
Female	14	35
Age (n = 40)		
11 years	7	17.5
12 years	33	82.5
BMI (n = 40)		
< 18.5	10	25.0
18.5–22.9	21	52.5
23.0–24.9	7	17.5
> 25	2	5.0
Parental smoking (n = 40)		
Smoking	18	45.0
Non-smoking	22	55.0
Parental income (n = 40)		
High	12	30.0
Middle	21	52.5
Low	9	22.5

### qPCR

The results of qPCR are shown in Table 4. No significant difference was noted among the four dietary pattern groups with regard to the number of total bacteria, *Bifidobacterium*, *Lactobacillus*, *Clostridium*, and *Escherichia coli* strains. However, the number of *Enterobacter* strains was significantly (*p* < 0.05) higher in the processed food group, and the ratio of *Lactobacillus* to *Enterobacter* strains was significantly (*p* < 0.05) lower in the processed food group than in the healthy group. The number of pathogenic bacteria, such as the *Clostridium* and *Enterobacter* strains, was significantly (*p* < 0.05) higher, whereas the ratio of *Lactobacillus* to *Enterobacter* strains was significantly (*p* < 0.05) lower in the ADHD group than in the control group.

**Table 4:** The counts (log10 16S rDNA gene copies g-1 of colonic digest) of different bacterial groups measured by quantitative real-time polymerase chain reaction (qPCR).

Bacteria	Dietary patterns					ADHD scores		
	Healthy	Processed food	Fish and shellfish	Meat	*p*-value	Control	ADHD	*p*-value
Total bacteria	11.40±0.31^1)^	11.41±0.21	11.24±0.23	11.17±0.21	NS^2)^	11.37±0.14	10.99±0.24	NS^4)^
*Bifidobacterium*	10.14±0.13	10.35±0.21	10.11±0.14	10.25±0.13	NS	10.16±0.08	10.41±0.15	NS
*Lactobacillus*	8.87±0.17	8.59±0.28	8.67±0.19	8.49±0.26	NS	8.67±0.12	8.70±0.29	NS
*Clostridium*	7.73±0.20	7.57±0.33	7.38±0.31	7.64±0.26	NS	7.39±0.13	8.51±0.16*	<0.05^5)^
*Enterobacter*	6.57±0.15^b^	7.53±0.19^a^	7.18±0.37^ab^	7.20±0.26^ab^	<0.05^3)^	6.91±0.13	7.84±0.28*	<0.05
*Escherichia coli*	6.21±0.16	6.95±0.36	6.75±0.33	6.84±0.23	NS	6.53±0.14	7.22±0.41	NS
*Lactobacillus*: *Enterobacter*	2.30±0.17^a^	1.06±0.19^b^	1.49±0.41^ab^	1.29±0.38^ab^	<0.05	2.09±0.14	0.86±0.30*	<0.05

^1)^ Values represent the mean ± standard error of the mean (SEM)

^2)^ NS, there were no significant differences by one-way analysis of variance (ANOVA) test

^3) abc^ Groups with different letters in the same row are significantly different by one-way ANOVA with Duncan’s multiple range test

^4)^ NS, there were no significant differences by Student’s t-test

^5)^ The statistical significance between the control and ADHD groups was analyzed by the Student’s t-test (*p < 0.05)

### Metagenomics analysis

**Fig 1:**
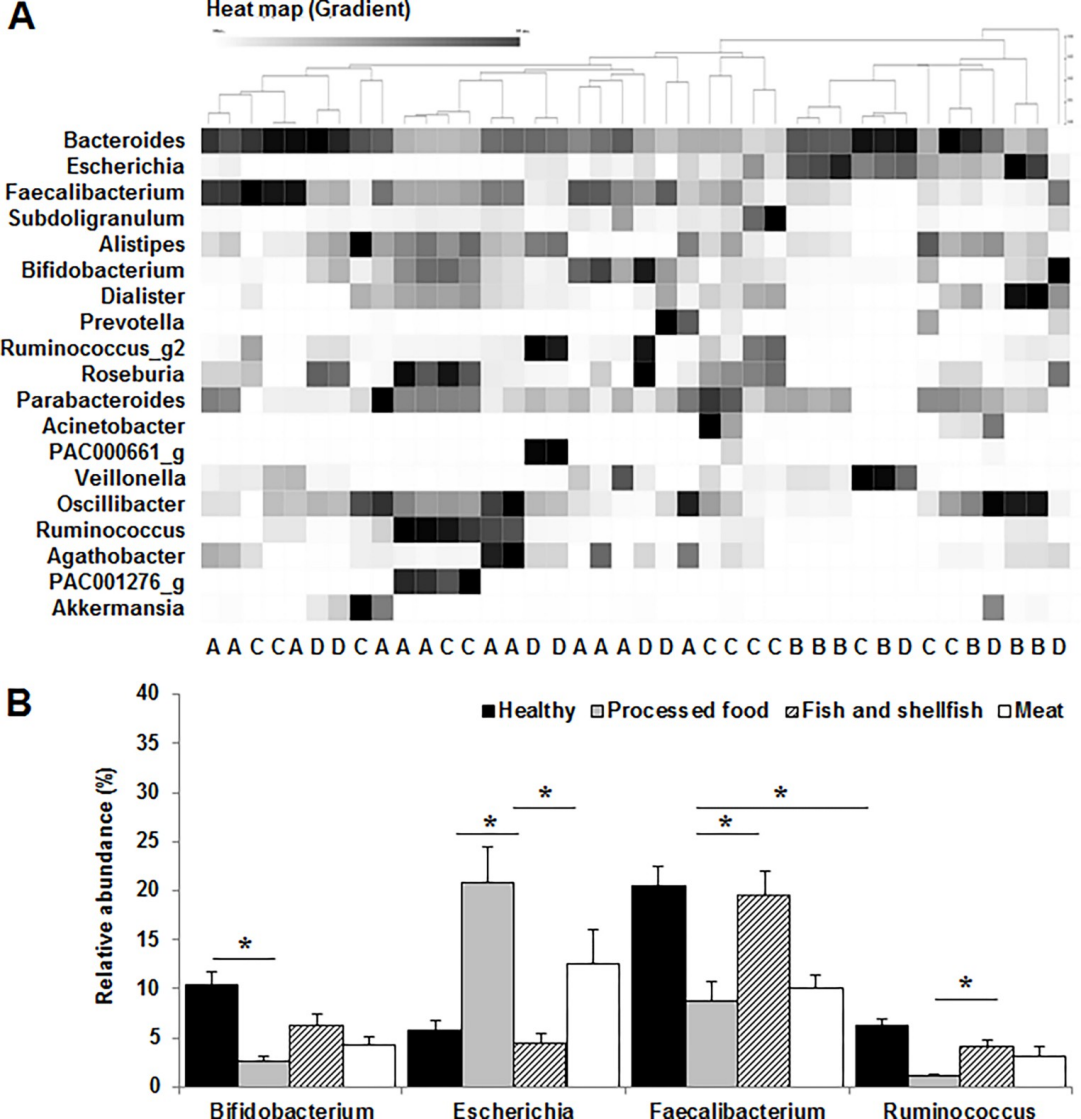
Comparison of the gut microbiota at the genus level according to the dietary patterns. (**A**) Heat map of different genera in the gut microbiota. A, healthy; B, processed food; C, fish and shellfish; D, meat. (**B**) Relative abundance of the gut microbiota. Values are expressed as the mean ± standard error of the mean (SEM). Statistical significance was determined by one-way analysis of variance (ANOVA) with Duncan’s multiple range test (*p < 0.05).

Metagenomic analysis was conducted to investigate the gut microbiota composition and diversity in the feces of the participants. Heat map analysis of different genera of the gut microbiota revealed that the microbiota profile of fish and shellfish dietary pattern and meat dietary pattern did not have distinct clusters, whereas that of the processed food dietary pattern mostly belonged to a separate cluster from that of the healthy group (Fig 1A). Moreover, the composition of the gut microbiota at the genus level according to the dietary pattern showed that the relative abundance of beneficial bacteria, such as the *Bifidobacterium*, *Faecalibacterium*, and *Ruminococcus* strains, was significantly (*p* < 0.05) lower in the processed food group or meat group, whereas that of harmful bacteria, such as *E*. *coli*, was markedly (*p* < 0.05) higher in the processed food group than in the healthy group (Fig 1B).

**Fig 2:**
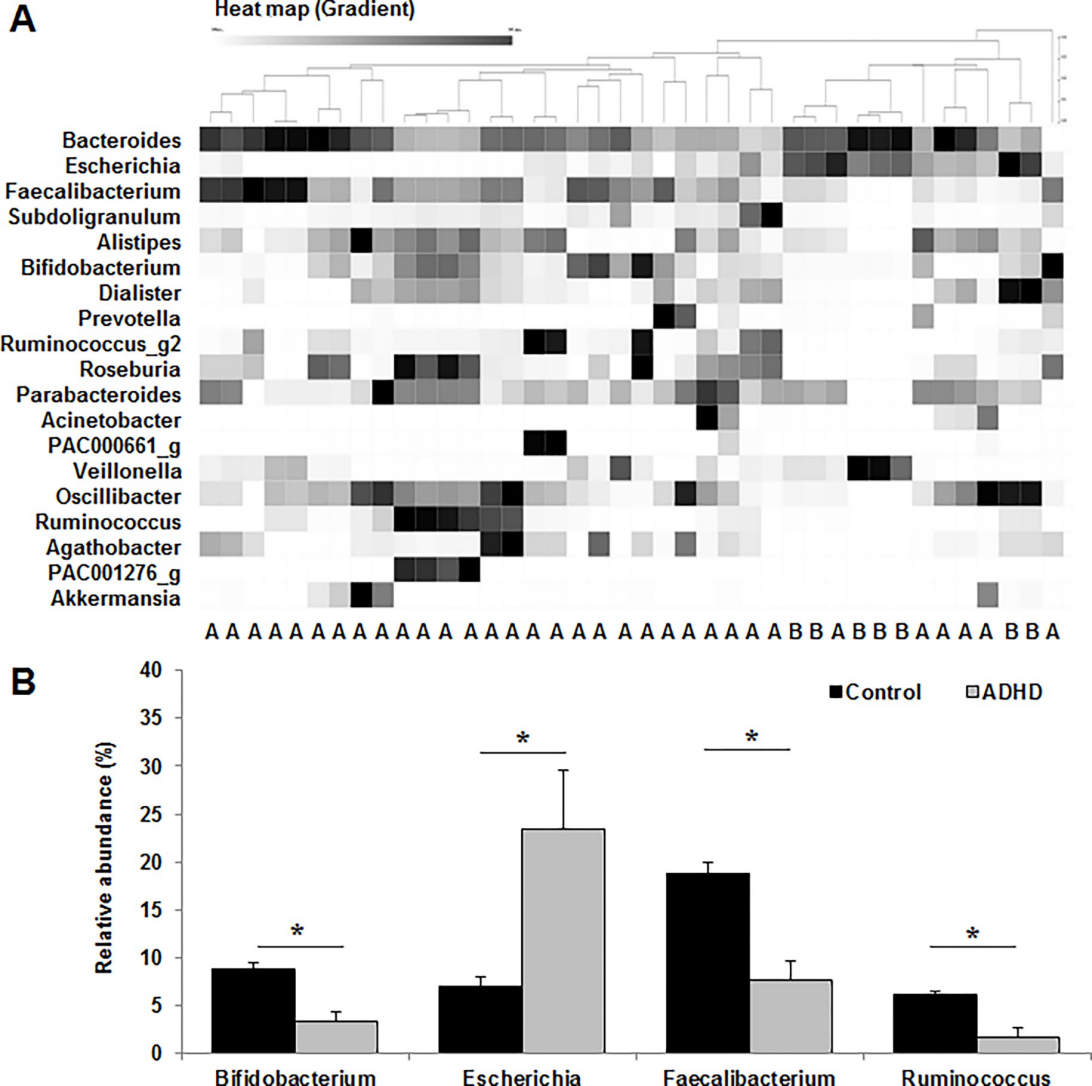
Comparison of the gut microbiota at the genus level between the control and attention deficit hyperactivity disorder (ADHD) groups. (**A**) Heat map of different genera in the gut microbiota. A, control group; B, ADHD group. (**B**) Relative abundance of the gut microbiota. Values are expressed as the mean ± SEM. The statistical significance between the control and ADHD groups was analyzed by the Student’s t-test (*p < 0.05).

Heat map analysis of the different genera of the gut microbiota according to ADHD score showed that the microbiota profiles of the control and ADHD groups mostly belonged to distinct clusters (Fig 2A). The relative abundance of harmful bacteria, such as the *Escherichia* strains, was significantly (*p* < 0.05) higher in the ADHD group, whereas that of beneficial bacteria, such as the *Bifidobacterium*, *Faecalibacterium*, and *Ruminococcus* strains, was markedly (*p* < 0.05) lower in the ADHD group than in the control group (Fig 2B).

**Fig 3:**
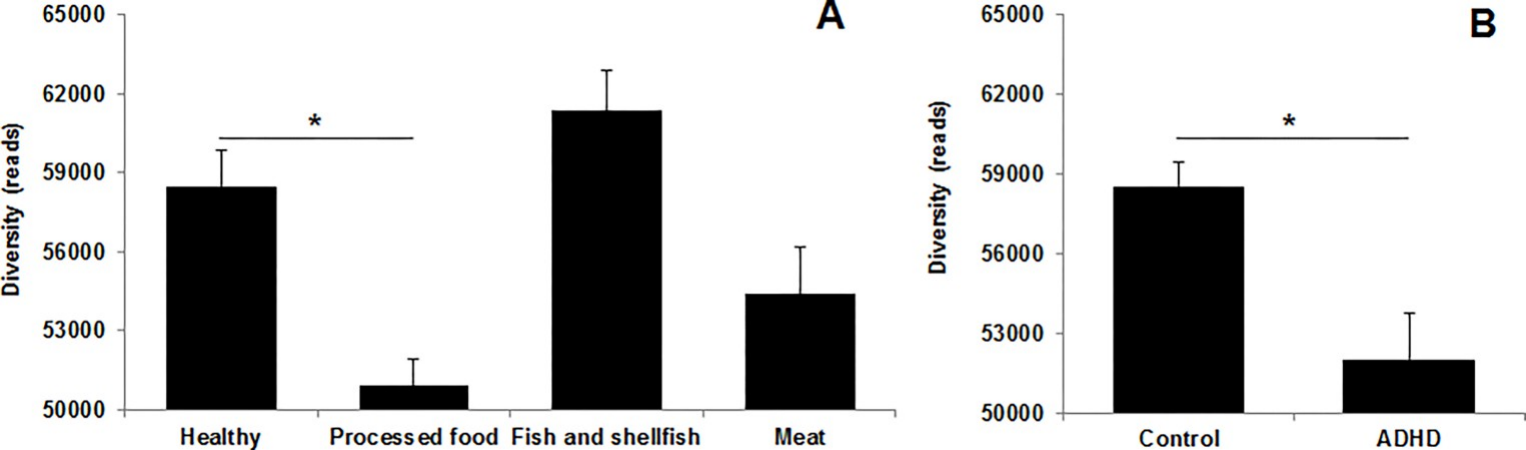
The α-diversity of the gut microbiota. (**A**) Dietary patterns; (**B**) ADHD scores. Values are expressed as the mean ± SEM. Statistical significance was analyzed by (**A**) one-way ANOVA with Duncan’s multiple range test (*p < 0.05) and (B) Student’s t-test (*p < 0.05).

The α-diversity of the processed food group was significantly lower (*p* < 0.05) than that of the healthy group (Fig 3A, and that of the ADHD high-risk group was markedly (*p* < 0.05) lower than that of the control group (Fig 3B).

The results of the correlation analysis for ADHD scores, dietary patterns, and gut microbiota composition are shown in Table 5. The processed dietary pattern was positively (*p* < 0.05) correlated with the relative abundance of *Escherichia* strains, whereas it was negatively (*p* < 0.05) associated with the α-diversity and composition of *Bifidobacterium*, *Faecalibacterium*, and *Ruminococcus* strains. Furthermore, the ADHD score was significantly (*p* < 0.05) associated with increased composition of *Escherichia* strains, whereas markedly (*p* < 0.05) was associated with decreased α-diversity and the relative abundance of *Bifidobacterium*, *Faecalibacterium*, and *Ruminococcus* strains. These results showed that processed food dietary patterns may increase ADHD scores, disrupting the balance and diversity of the gut microbiota.

**Table 5:** Correlation analysis among the dietary pattern, attention deficit hyperactivity disorder (ADHD) score, and gut microbiota composition.

	AD score	HD score	Healthy	PF	FS	Meat	Diversity	BIF	ESCH	FAE	RUM
AD score	1										
HD score	.975**	1									
Healthy	-.309	-.314	1								
PF	.387*	.372*	-.046	1							
FS	-.047	-.059	-.050	.003	1						
Meat	.266	.264	-.064	-.029	.001	1					
Diversity	-.370*	-.378*	.255	-.388*	.037	-.225	1				
BIF	-.338*	-.370*	.234	-.322*	-.228	-.112	.300	1			
ESCH	338*	319*	-.214	.380*	-.008	.208	-.422**	-.459**	1		
FAE	-.360*	-.364*	.094	-.347*	-.092	-.233	.412**	.322*	-.667**	1	
RUM	-.341*	-.371*	.288	-.315*	.087	-.160	.456**	.437**	-.642**	.461**	1

AD, attention deficit; HD, hyperactivity disorder; PF, processed food; FS, fish and shellfish; BIF, *Bifidobacterium*; ESCH, *Escherichia*; FAE, *Faecalibacterium*; RUM, *Ruminococcus*. Correlation analysis was performed using the Pearson correlation coefficient (*p < 0.05 or **p < 0.01)

### Fecal short-chain fatty acids

The fecal SCFAs of the participants were measured by gas chromatography. No significant decrease in the concentration of propionate was noted between the control and experimental groups (Table 6). However, the concentration of fecal butyrate was markedly (*p* < 0.05) lower in the processed food group than in the healthy group, and the ADHD group showed significantly (*p* < 0.05) lower concentrations of fecal acetate and butyrate than those of the control group. The analysis of correlation among ADHD score, dietary patterns, and SCFA concentration in feces is shown in Table 7. The processed dietary pattern was negatively (*p* < 0.05) correlated with the concentration of butyrate, and ADHD score was markedly (*p* < 0.05) associated with a decrease in the concentration of butyrate, compared to the control group.

**Table 6:** Concentrations of short-chain fatty acids in the feces of Korean elementary school students according to their dietary patterns and ADHD scores.

Fatty acids (mmol/g)	Dietary patterns					ADHD scores		
	Healthy	Processed food	Fish and shellfish	Meat	*p*-value	Control	ADHD	*p*-value
Acetic acid	6.44±0.15^1)^	6.05±0.17	6.21±0.15	5.89±0.16	NS^2)^	6.34±0.09	5.73±0.07*	< .05^4)^
Propionic acid	4.44±0.20	4.25±0.12	4.45±0.08	4.37±0.12	NS	4.42±0.08	4.27±0.11	NS^5)^
Butyric acid	3.91±0.05^a^	3.42±0.07^b^	3.87±0.10^a^	3.67±0.09^ab^	< .05^3)^	3.96±0.03	3.60±0.6*	< .05

^1)^ Values are represented as the mean ± SEM

^2)^ NS, There were no significant difference by one-way ANOVA test

^3) abc^ Groups with different letters in the same row are significantly different by one-way ANOVA with Duncan’s multiple range test

^4)^ The statistical significance between the control and ADHD groups was analyzed by the Student’s t-test (*p < 0.05)

^5)^ NS, there were no significant differences by Student’s t-test

**Table 7:** Correlation analysis among the dietary patterns, ADHD scores, and the concentrations of short-chain fatty acids.

	AD score	HD score	Healthy	PF	FS	Meat	AA	PA	BA
AD score	1								
HD score	.975	1							
Healthy	-.309	-.314	1						
PF	.387*	.372*	-.046	1					
FS	-.047	-.059	-.050	.003	1				
Meat	.266	.264	-.064	-.029	.001	1			
AA	-.287	-.274	.268	-.185	-.204	.020	1		
PA	-.104	-.110	-.035	-.065	.008	.177	.308	1	
BA	-.368*	-.346*	.286	-.365*	-.114	-.031	.556**	.314	1

AD, attention deficit; HD, hyperactivity disorder; PF, processed food; FS, fish and shellfish; AA, acetic acid; PA, propionic acid; BA, butyric acid. Correlation analysis was performed using the Pearson correlation coefficient (*p < 0.05 or **p < 0.01)

## Discussion

ADHD Rating Scale (ARS)-VI is a tool developed to screen ADHD for children and adolescents aged 5 to 18 [31]. The ARS-VI was consisted of nine question related to the attention deficit and hyperactivity disorders, respectively. Each question had a four-point answer format (0 = never, 1 = sometimes, 2 = often, 3 = very often). We evaluated ADHD scores of elementary school students using K-ARS, which is the Korean version of ARS-VI. The K-ARS have been generally using in research related to ADHD in Korea to screen children’s ADHD, and both attention deficit and hyperactivity disorders were also found a good internal consistency. In this study, we classified the dietary patterns of participants into four categories using a dish-based semi-quantitative FFQ and Can Pro 4.0 (The Korean Nutrition Society) database. Four dietary patterns were found good validation, and explained 44.56% of the variance in food intake. A validation study on our dietary patterns can be also found elsewhere [27, 28]. Therefore, in this study, we guess that measurements for ADHD and dietary patterns are valuable when compared to other studies. However, there is a limitation in that the ADHD score was assessed by their parents, the correlation between ADHD symptoms and dietary patterns can be biased to invalid as participants’ parents may underestimate ADHD symptoms.

In this study, participants who consume probiotics, antibiotics, and health supplements were excluded from the survey, and we investigated the basic characteristics of participants, such as sex, age, BMI, parental smoking, parental income. The basic characteristics of participants did not significantly relate to the dietary patterns and ADHD scores. However, the covariates/confounders may not be sufficiently considered, resulting in bias of the research results.

Dietary intake plays an important role in the modulation of gut microbiota and brain neurotransmission. Mental disorders are regulated by the function of the brain and an imbalance of the gut microbiota composition is likely to increase the incidence of mental disorders, such as ADHD [29]. Some studies have indicated that dietary patterns are associated with ADHD symptoms [26] and we identified four major dietary patterns (healthy, processed food, fish and shellfish, and meat), explaining 44.65% of the variation in this study. This value was similar to that reported in other studies [26, 28, 32]. Healthy, shellfish, and meat dietary patterns were not statistically correlated with ADHD scores. However, processed food dietary patterns, which are characterized by high intake of fats, sweets, and fast foods, were positively correlated with the increase in ADHD score. Fast foods and sweet dietary patterns have been reported to be associated with an exacerbation of ADHD symptoms owing to the high intake of saturated fat and refined sugar [25, 32] and we found that saturated fat was positively correlated to processed food dietary patterns as well as ADHD scores. A diet high in saturated fat may reduce the relative abundance of *Alistipes* strains, which are known to produce butyrate [33], and enhance the growth of harmful bacteria, such as the *Enterobacter* strains [34]. In addition, Zhang and Yang [35] reported that a high-saturated fat diet can decrease gut microbiota diversity, resulting in gut microbiota dysbiosis. Processed food dietary patterns can induce higher intake of saturated fat, which contributes to gut microbiota dysbiosis; these alterations may aggravate ADHD symptoms.

The composition of the gut microbiota plays an important role in the maintenance of health by regulating metabolites, such as fecal SCFAs, which can protect the host from external pathogens and potentially harmful bacteria residing in the intestine [36]. The results of qPCR revealed that the processed food dietary pattern was markedly associated with an increased number of *Enterobacter* strains, whereas the ratio of *Lactobacillus* to *Enterobacter* strains was significantly lower than that of the healthy group. Moreover, the number of harmful bacteria, such as the *Clostridium* and *Enterobacter* strains, was considerably higher, whereas the ratio of *Lactobacillus* to *Enterobacter* strains was significantly lower in the ADHD group than in the control group. The ratio of *Lactobacillus* to *Enterobacter* strains is known as an index of a healthy intestine, and a high index indicates improvement in intestinal protection against opportunistic pathogens [37]. A variety of studies have shown that the relative abundance of harmful bacterial strains was higher in children with psychological problems than in healthy children [38–40].

Heat map analysis of similar clusters analyzed using the CLcommunity program showed that the dietary intake was closely associated with the alteration of the gut microbiota composition, and these changes were further correlated with the ADHD score. The gut microbiota profile of the processed food and ADHD groups mostly belonged to distinct clusters, unlike that of the control group. The relative abundance of *Bifidobacterium*, *Faecalibacterium*, and *Ruminococcus* strains, which are closely associated with ADHD, were significantly lower in the processed food group and ADHD group than in the control group. Beneficial bacteria, such as the *Bifidobacterium* strains, modulate the balance of gut microbiota composition and interfere with the toxicity of pathogenic strains and may have beneficial effects on psychological health [41, 42]. It was also reported that the relative abundance of *Faecalibacterium* and *Ruminococcus* strains was markedly reduced in children with ADHD [43]. Moreover, *Faecalibacterium* strains can induce the production of SCFAs [44], which contribute to the modulation of the intestinal environment to facilitate the growth of beneficial bacteria, such as the *Bifidobacterium* strains [22]. This can contribute to the prevention and improvement of disease through the balanced composition of the gut microbiota. The processed food and ADHD groups had significantly reduced α-diversity of fecal microbiota compared to that of the control group, and the α-diversity of gut microbiota composition tended to decrease in children with ADHD [45].

The gut microbiota can synthesize neurotransmitters, such as dopamine [46], which can regulate the attention, cognition, memory, learning, and emotional behavior, and Ayano [16] reported that the reduction of dopamine in the brain was associated with the exacerbation of ADHD symptoms. The balance of gut microbiota composition plays an important role in inducing the synthesis of dopamine, whereas the collapse of a well-balanced gut microbiota may interfere with the production of dopamine [47].

The concentration of fecal SCFAs was altered depending on the dietary pattern or ADHD score. No significant difference in propionate concentration of feces was noted between the control and experimental groups; however, the processed food group and the ADHD group showed a marked decrease in the concentration of acetate or butyrate compared to that in the control group. Fecal SCFAs, such as acetate and butyrate, can regulate intestinal homeostasis and have a direct effect on the gut microbiota composition [21]. Several studies have shown that fecal SCFAs inhibit the growth of pathogenic bacteria, such as the *Salmonella*, *E*. *coli*, and *Clostridium* strains [48, 49]. In addition, butyrate plays an important role in the modulation of neuronal excitability and ENS activity [50], and Dalile et al. [51] reported that fecal SCFAs play a key role in the interaction of microbiota-gut-brain. Fecal SCFAs are recognized by receptors, such as enteroendocrine and enterocyte cells, and they can modulate brain function through the synthesis of neurotransmitters [47]. The combination of fecal SCFAs and receptors stimulates signaling to the brain through the vagal pathway by inducing the secretion of neurotransmitters, such as serotonin, which is known to be reduced in patients with ADHD [17]. Through which fecal SCFAs may be able to affect cognition and emotional functions of the brain of patients with ADHD.

Our study several limitations should be considered. First, the number of samples is insufficient, and there is a limitation in that the evaluation of ADHD was conducted by their parent-assessment, not by a doctor’s diagnosis. The association between ADHD symptoms and dietary patterns can be biased to invalid as participants may underestimate ADHD symptoms. Second, there is a limitation in that bias may occur in the research results because covariates for the characteristics of the study participants are not sufficiently considered. Future studies need to increase the number of samples and analyse the stronger association between ADHD and dietary patterns using other methods of evaluating ADHD.

In summary, we analyzed the dietary patterns and ADHD scores of 40 school-age children using FFQ or K-ARS. The feces of the participants were collected to investigate the composition of the gut microbiota and SCFAs. The dietary patterns of candidates were divided into four groups: healthy, processed food, fish and shellfish, and meat. The participants were also separated into two groups according to their ADHD scores: control or ADHD group. The ADHD score of the processed food group was significantly higher than that of the healthy group. The abundance of harmful bacteria, such as *Enterobacteria*, *E*. *coli*, and *Clostridium*, was considerably higher, while that of beneficial bacteria, such as the *Bifidobacterium* and *Ruminococcus* strains, was remarkably lower in the processed food, meat, and ADHD groups than the control group. Therefore, the finding that the processed food and ADHD groups showed remarkably reduced α-diversity of gut microbiota and significantly reduced concentrations of acetic acid or butyric acid in the feces compared to the control group. Since our study has limitations according to the number of samples and the ADHD assessment method, it is necessary to conduct research through connection with hospitals in the future to derive more meaningful results.
